# Bronchiectasis in Adults: Aetiology and New Therapies

**DOI:** 10.3390/jcm11195957

**Published:** 2022-10-09

**Authors:** Paul T. King, Lucy Morgan

**Affiliations:** 1 Monash Lung, Sleep, Allergy and Immunology, Monash Medical Centre (MMC), Melbourne 3168, Australia; 2 Department of Medicine, Monash Medical Centre (MMC), Monash University, Melbourne 3168, Australia; 3 Department of Respiratory Medicine, Concord Hospital, Sydney 2138, Australia; 4 Faculty of Medicine, University of Sydney, Sydney 2006, Australia

Bronchiectasis is emerging as a global health issue, and this is reflected by a series of registries that were established worldwide [1,2,3,4]. In addition, a significant proportion of subjects with chronic obstructive pulmonary disease (COPD) have co-existent bronchiectasis, which is associated with more severe disease and poorer outcomes [5]. However, despite its prevalence and importance, the pathogenesis and optimal treatment of this condition are still not well defined. This Special Issue in the journal highlights new insights into the aetiology and treatment of adults with bronchiectasis.

Bronchiectasis is a very heterogeneous condition with a large variety of potential causes and risk factors. It has been recognised for many years that tuberculosis (TB) infection may result in the development of bronchiectasis; however, there have been minimal published studies that have characterised this important entity. Choi et al. described a cohort of 118 post-TB patients derived from the Korean registry of 598 subjects [6]. The post-TB patients generally had worse disease with lower body mass index and lung function and more extensive involvement in CT scanning with prevalent upper lobe changes. The authors speculated that bronchodilator usage might be helpful in post-TB patients; however, this subgroup did not appear to have any evidence of significant bronchodilator response on lung function testing, and the evidence for the effectiveness of these medications in bronchiectasis is limited. Interestingly, this study described that post-TB disease was associated with a higher incidence of nontuberculous mycobacteria (NTM) colonisation.

NTM infection in bronchiectasis is important but not well understood. Dettmer and colleagues described computed tomography (CT) findings in 36 adults with NTM compared to 92 subjects without NTM pulmonary disease [7]. Patients with NTM infection had distinctive features on CT scanning of right middle lobe disease, extended bronchiolitis and more prominent nodular and cavitary expression. These radiologic features highlighted the likely presence of NTM infection. The implication of this work is that CT scans in patients with bronchiectasis should be carefully analysed by expert thoracic radiologists for underlying aetiologic causes, which may guide appropriate future management (e.g., the requirement for bronchoscopy).

As described by Peter Cole, the pathogenesis of bronchiectasis arises from a persistent infection that drives airway inflammation to become a self-perpetuating process, leading to lung damage (“the vicious cycle”) [8]. This has been most clearly defined in patients with more severe disease. In this Special Issue, systemic inflammation and redox balance were studied in patients with mild bronchiectasis [9]. The cohort studied was fairly small, with only 30 subjects compared to 26 well-matched controls, but otherwise a well-done and comprehensive study. Subjects with bronchiectasis had significantly higher levels of acute phase reactants, neutrophils, immunoglobulins and markers of oxidative stress (redox) balance. Whilst the findings were not surprising, this study does emphasise that subjects with mild disease clearly have established systemic inflammation, and early targeted therapy could potentially alter long-term outcomes.

Bronchiectasis has a wide range of outcomes, but once the disease is established, patients tend to have persistent disease [8] and may have a gradual decline in lung function [10]. Qin et al. described outcomes in a cohort of subjects with bronchiectasis followed up for one year into the COVID-19 pandemic in Spain [11], a country with extensive lockdowns during this period. This pilot study of 30 patients followed up for 12 months demonstrated a significant decline in lung function and increased exacerbation numbers and severity scores. They reported there was a significant decrease in inflammatory markers; there was no clear explanation given for this surprising finding. The authors did not appear to investigate any correlation between COVID lockdowns and worse outcomes; but decreased access to medical services could potentially have had an adverse effect on patient care. In general, it appears that infection with SARS-CoV-2 is not associated with a high incidence of bacterial infection, but there is a lack of literature on the effect of COVID-19 pandemic in bronchiectasis. This is an important area for future research [12].

There have been few randomised therapeutic trials in bronchiectasis, and subsequently, optimal management remains relatively ill-defined, although larger studies such as those of the European Multicentre Bronchiectasis Audit and Research Collaboration (EMBARC) are changing this paradigm. With antibiotics, sputum clearance/chest physiotherapy remains a cornerstone of patient management, although there is a range of different techniques utilised [13]. A major consequence of lockdowns in the COVID-19 pandemic was the initiation of telehealth. Remote assessment is particularly challenging for physiotherapy, in which physical communication and touch have a key role. Lee and colleagues assessed a telehealth model of physiotherapy with prospective follow-up using semi-structured interviews [14]. This study only included nine participants with overall mixed opinions of the benefit of this intervention compared to traditional face-to-face interaction. There was an acceptance of this model as required by circumstances, but with an ongoing preference for in-person consultations by some participants. Chest physiotherapy is still limited in its availability, so some form of telehealth for selected patients may improve this situation in the future.

This Special Issue on bronchiectasis describes some important new developments in the field. Mycobacterial infections are an important but neglected area with specific ramifications for patient outcomes and treatment. The likelihood of ongoing COVID-19 outbreaks and the need for telehealth are new issues that warrant further investigation.
